# Involvement of the neuronal phosphotyrosine signal adaptor N-Shc in kainic acid-induced epileptiform activity

**DOI:** 10.1038/srep27511

**Published:** 2016-06-08

**Authors:** Shiro Baba, Kazuko Onga, Sho Kakizawa, Kyoji Ohyama, Kunihiko Yasuda, Hiroshi Otsubo, Brian W. Scott, W. McIntyre Burnham, Takayuki Matsuo, Izumi Nagata, Nozomu Mori

**Affiliations:** 1 Department of Neurosurgery, Nagasaki University Graduate School of Biomedical Sciences, Nagasaki, Japan; 2 Department of Anatomy and Neurobiology, Nagasaki University Graduate School of Biomedical Sciences, Nagasaki, Japan; 3 Division of Neurology, The Hospital for Sick Children, Toronto, ON, Canada; 4 Department of Pharmacology and Toxicology and the University of Toronto Epilepsy Research Program, Faculty of Medicine, University of Toronto, Toronto, ON, Canada

## Abstract

BDNF-TrkB signaling is implicated in experimental seizures and epilepsy. However, the downstream signaling involved in the epileptiform activity caused by TrkB receptor activation is still unknown. The aim of the present study was to determine whether TrkB-mediated N-Shc signal transduction was involved in kainic acid (KA)-induced epileptiform activity. We investigated KA-induced behavioral seizures, epileptiform activities and neuronal cell loss in hippocampus between N-Shc deficient and control mice. There was a significant reduction in seizure severity and the frequency of epileptiform discharges in N-Shc deficient mice, as compared with wild-type and C57BL/6 mice. KA-induced neuronal cell loss in the CA3 of hippocampus was also inhibited in N-Shc deficient mice. This study demonstrates that the activation of N-Shc signaling pathway contributes to an acute KA-induced epileptiform activity and neuronal cell loss in the hippocampus. We propose that the N-Shc-mediated signaling pathway could provide a potential target for the novel therapeutic approaches of epilepsy.

Epilepsy is a brain disorder with a variable age-adjusted prevalence ranging from 0.4 to 0.8%^1^. Approximately 20% of patients with epilepsy have seizures that are not adequately controlled by antiepileptic drugs(AEDs)^2^. It is commonly assumed that an imbalance between the excitation and inhibition in the brain initiates seizure activity, however, the molecular mechanisms underlying seizure activity are poorly understood. Elucidating the molecular mechanisms of epileptiform activity would provide insights which could lead to the development of novel therapeutic approaches for epilepsy.

Chemo-convulsants, such as kainic acid(KA), have been widely used to study the basic mechanisms involved in temporal lobe epilepsy(TLE) and seizures, and to evaluate the efficacy of AEDs. TLE is often associated with neuronal cell loss in the hippocampus, i.e., hippocampal sclerosis. KA treatment of animals causes depolarization of neurons, behavioral seizures, status epilepticus and also neuronal cell death in the hippocampus. This leads to spontaneous seizures that are considered an animal model for TLE of human^3^.

Using the KA-induced epilepsy paradigm, many studies have demonstrated that brain-derived neurotrophic factor(BDNF) and its receptor tropomyosin-related kinase B(TrkB) play critical roles in seizures and epileptogenesis. The expression of BDNF is massively induced following seizures, and the TrkB receptor is activated in the hippocampus of various animals models of seizures^4^,^5^,^6^,^7^. Inhibition of TrkB commencing after KA induced status epilepticus prevented recurrent seizures and limited loss of hippocampal neuron^8^.

BDNF not only enhances excitatory synaptic transmission but also reduces GABAergic inhibitory synaptic transmission^9^. The activation of TrkB reduces the expression of the K^+^-Cl^−^-cotransporter2(KCC2) and impairs Cl^−^ extrusion, thereby reducing GABA_A_ receptor-mediated synaptic inhibition^10^, and leading to an imbalance in synaptic transmission in hippocampal neural networks^7^. Taken together, these data suggest that an aberrant activation of BDNF-TrkB signaling might underlie the initiation of epileptiform activities and seizures.

At the molecular level, activation of the TrkB receptor by BDNF requires protein dimerization and the subsequent autophosphorylation of tyrosine residues within the intracellular domain of the TrkB receptor. The phosphorylated tyrosine residues are recognized and bound by docking and/or adaptor proteins, such as Grb2, Shc, and PLCγ^11^. Increase of complex level of interaction between TrkB and Shc after BDNF treatment was observed using cellular BRET assay^12^. We have previously isolated the neural-specific phosphotyrosine signal adaptor Shc, which is also called neuronal Shc(N-Shc)^13^. The expression of N-Shc correlates with neuronal differentiation and maturation in the central nervous system. Thus, N-Shc plays critical roles in BDNF-TrkB signal transduction and NMDA function^14^,^15^,^16^.

A point mutation in the Shc binding site of TrkB was studied for kindling mice^17^. Disruption of TrkB-mediated activation of PLCγ signaling inhibited kindling and KA-induced spontaneous seizures^18^,^19^. However, a role of N-Shc in TrkB-mediated signal transduction have never been studied in the KA-induced seizures. The present study was designed to provide the role of N-Shc, downstream signal adaptor of TrkB receptor, in KA-induced seizures. We hypothesize that N-Shc-mediated signaling pathway is related to epileptiform activity and that the suppression of N-Shc can reduce seizures. We therefore used N-Shc deficient mice to examine the potential role for N-Shc in KA-induced epileptiform activity.

## Results

### Expressions of TrkB and KA-related receptors are unaffected in N-Shc deficient mice

Before testing the N-Shc−/− mice with KA, we wanted to confirm that N-Shc protein was decreased in the mutant mice, and also to determine whether the expression of the TrkB and KA receptors (i.e., GluR6, KA1, and KA2) was normal.

Western blot analyses of protein extracts from several brain regions - cerebral cortex, hippocampus, and thalamus - were done in control (n = 3) and N-Shc−/− mice (n = 3).

**Figure 1:**
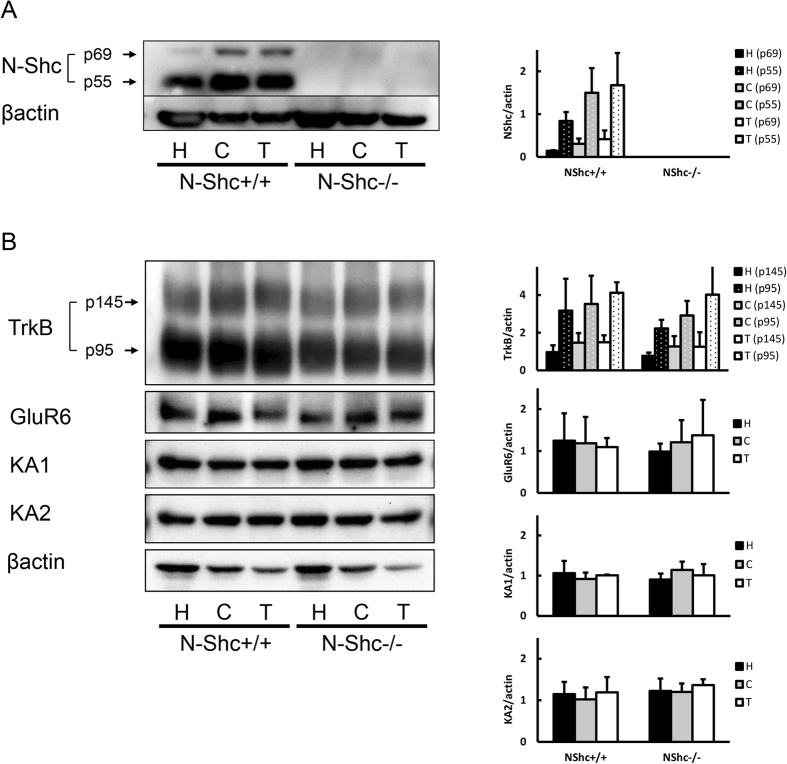
Western blot analyses of the brains isolated from either control or N-Shc−/− mice. The expression of two isoforms of the N-Shc protein (p69, p55) was measured in tissue extracts of the hippocampus (H), cerebral cortex (C) and thalamus (T) from the control and mutant mice. In contrast to controls, N-Shc−/− mice showed a complete loss of the two isoforms of N-Shc protein (**A**). The expression levels of TrkB, GluR6, KA1 and KA2 also did not show significant differences between the control and N-Shc−/− mice (**B**).

It was found that protein extracts from the control mice contained both the large and small isoforms of N-Shc protein, i.e., p69 and p55, in all brain regions, whereas protein extracts from the N-Shc−/− mice revealed no corresponding bands for the N-Shc proteins (p < 0.05, Fig. 1A). The expression of the various receptors related to Shc signaling, i.e., TrkB (p145 full length form and p95 truncated form) and of the various subtypes of kainate receptors (GluR6, KA1, KA2), however, was not significantly different between the control and N-Shc−/− mice in any brain area (p > 0.05, Fig. 1B). We therefore conclude that the N-Shc deletion does not influence TrkB and KA-related receptor expression in the brain.

### KA-induced behavioral seizures are reduced in N-Shc deficient mice

We next examined KA-induced behavioral seizures in the N-Shc−/− (n = 22), control C57BL/6 (n = 24) and N-Shc+/+ mice (n = 18). KA was administered systemically (30mg/kg,i.p.) and the behavioral seizures were monitored for 320 minutes.

**Figure 2:**
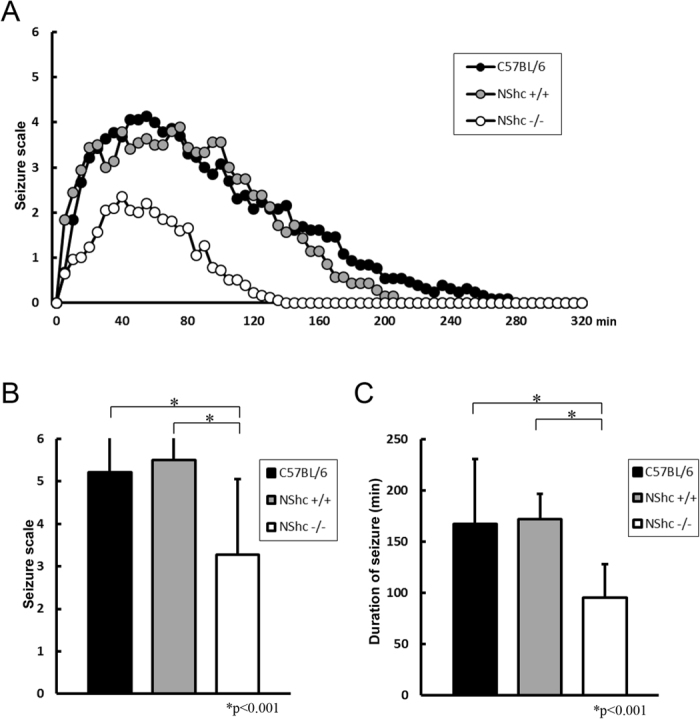
Inhibition of KA-induced behavioral seizures in N-Shc−/− mice. Time course of the behavioral seizure scores and the maximum seizure score after an intraperitoneal administration of KA. The mean maximum seizure score induced by KA administration was significantly lower in N-Shc−/− mice as compared with C57BL/6 and N-Shc+/+ mice (C57BL/6 mice n = 24, 5.21 ± 0.98; N-Shc+/+ mice n = 18, 5.5 ± 0.71; N-Shc−/− mice n = 22, 3.27 ± 1.78; p < 0.001) (**A,B**). The mean duration of the behavioral seizures was also significantly decreased in N-Shc−/− mice (C57BL/6 mice, 167.2 ± 63.8 min; N-Shc+/+ mice, 172.1 ± 24.6 min ; N-Shc−/− mice, 95.3 ± 32.6 min; P < 0.001) (**C**).

The N-Shc−/− mice exhibited decreased seizure severity as compared with the control C57BL/6 and N-Shc+/+ mice (Fig. 2A). The mean maximum seizure score after KA administration was significantly lower in the N-Shc−/− mice than in the control C57BL/6 and N-Shc+/+ mice (C57BL/6 mice, 5.21 ± 0.98; N-Shc+/+ mice, 5.50 ± 0.71; N-Shc−/− mice, 3.27 ± 1.78; p < 0.001) (Fig. 2B, Table 1). The mean duration of the behavioral seizures after KA administration was also significantly decreased in the N-Shc−/− mice as compared with the C57BL/6 and N-Shc+/+ mice (C57BL/6 mice, 167.2 ± 63.8 min; N-Shc+/+ mice, 172.1 ± 24.6 min; N-Shc−/− mice, 95.3 ± 32.6 min; P < 0.001) (Fig. 2C, Table 1). These results indicate that the N-Shc−/− mice are resistant to KA-induced behavioral seizures.

### Epileptiform discharges are reduced in the hippocampus of N-Shc deficient mice

We next examined electrographic seizure activity in hippocampus of C57BL6 (n = 5) and N-Shc−/− mice (n = 4). Seven days after implantation, the electrodes were connected to a lead, and digital EEG was recorded for 30 min before and for 120 min after KA administration.

**Figure 3:**
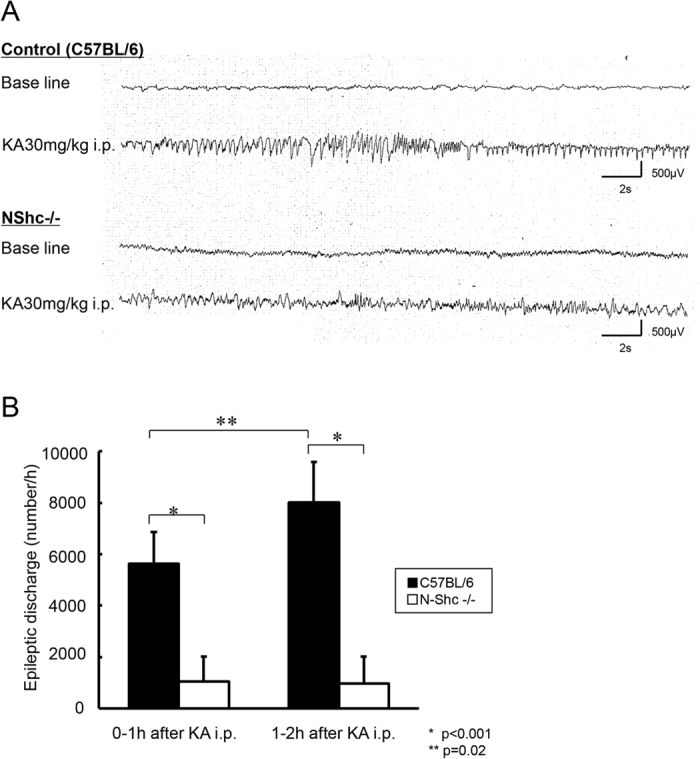
The epileptiform discharges recorded by hippocampal EEG were significantly reduced in N-Shc−/− mice. An example of the hippocampal EEG recordings registered before KA administration (base line) and during the behavioral seizures after KA administration (**A**). There were no obvious epileptiform discharges observed before the KA administration in either control C57BL/6 mice or N-Shc−/− mice. After KA administration, the frequency of the epileptiform discharges was significantly decreased in N-Shc−/− mice as compared with those in C57BL/6 mice within 1 hour (C57BL/6 mice n = 5, 5642.8 ± 1210 times/h; N-Shc−/− mice n = 4, 1046 ± 971 times/h; p < 0.001) and 1 ~ 2 hours (C57BL/6 mice, 8040 ± 1573 times/h; N-Shc−/− mice, 981.5 ± 1048 times/h; p < 0.001) (**B**). The epileptiform discharges in the C57BL/6 mice increased at 1–2 h after KA administration, whereas those in the N-Shc−/− mice were almost unchanged (p = 0.02) (**B**).

There were no obvious epileptiform discharges, such as spikes or sharp waves, before KA administration (Fig. 3A). After the administration of KA, epileptiform discharges appeared and continued for the whole period of observation. The amplitude and frequency of the epileptiform discharges, however, were lower in the N-Shc−/− mice (Fig. 3A). Similarly, the discharge frequencies were significantly lower for the NShc−/− mice (Fig. 3B). These data suggest that following KA treatment, the epileptiform discharges from the hippocampal neurons are significantly reduced in N-Shc−/− mice.

### Seizure-induced hippocampal neuronal cell loss is ameliorated in N-Shc deficient mice

We had previously reported that no gross structural abnormalities were observed in hippocampal of the N-Shc−/− mice on Nissl staining as compared to the wild-type mice^16^.

In the present study, we examined whether KA induced seizures caused morphological changes in the hippocampal regions of the C57BL/6 (day1, n = 6; day7, n = 4), N-Shc+/+ (day1, n = 3; day7, n = 3), and N-Shc−/− mice (day1, n = 7; day7, n = 6).

**Figure 4:**
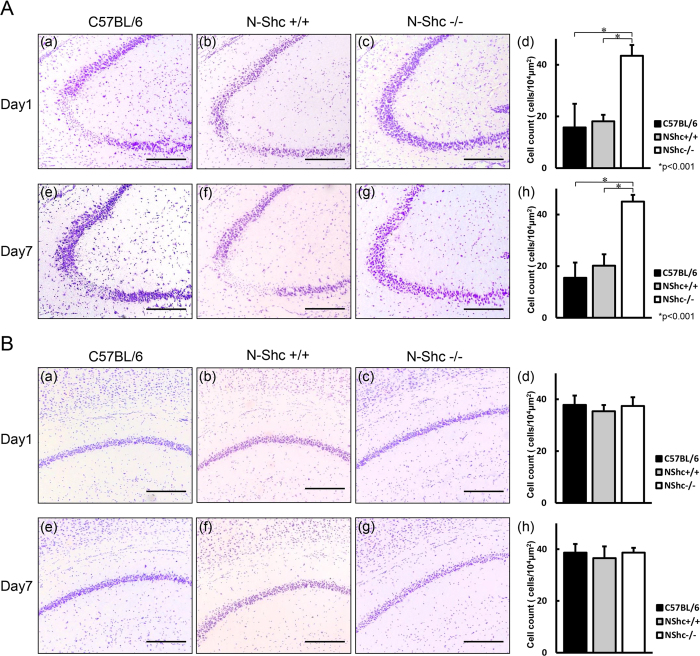
Reduction of KA-induced neuronal cell loss in the CA3 area of the hippocampus in N-Shc−/− mice. Coronal brain sections from C57BL/6, N-Shc+/+ and N-Shc−/− mice were processed by Nissl staining on 1 and 7 days after KA administration. C57BL/6 mice (a) day1 n = 6, (e) day7 n = 4; N-Shc+/+ mice (b) day1 n = 3, (f) day7 n = 3; and N-Shc−/− mice: (c) day1 n = 7, (g) day7 n = 6. Neuronal cell loss was observed in the pyramidal cell layer of the CA3 area of the hippocampus in C57BL/6 and N-Shc+/+ mice on day1 (**A**–a,b) and day7 (**A**–e,f) after KA administration. In contrast, neuronal cell loss in the CA3 area was prevented in N-Shc−/− mice on day1(**A**–c) and day7(**A**–g). Number of pyramidal cells in the CA3 area was significantly reduced in C57BL/6 and N-Shc+/+ mice as compared with those in N-Shc−/− mice (15.7 ± 9.1, 18.1 ± 2.5 and 43.5 ± 4.2 cells/10^4^ μm^2^ on day1; 15.5 ± 5.9, 20.2 ± 4.4, and 45.0 ± 2.6 cells/10^4^ μm^2^ on day7; respectively, p < 0.001) (**A**–d,h). Minimal, or no neuronal cell loss were observed in the CA1 area of the hippocampus in three groups on day1 (**B**–a,b,c) and day7 (**B**–e,f,g) after KA administration. Number of pyramidal cells in the CA1 area of the hippocampus showed no significant difference among C57BL/6, N-Shc+/+ and N-Shc−/− mice (37.8 ± 3.6, 35.4 ± 2.4, and 37.4 ± 3.4 cells/10^4^ μm^2^ on day1; 38.7 ± 3.3, 36.5 ± 4.5, and 38.6 ± 1.9 cells/10^4^ μm^2^ on day7; respectively, p > 0.05) (B-d, h). Scale bars = 200 μm.

On day 1 after KA, the number of pyramidal cells in the CA3 region was 15.7 ± 9.1 cells/10^4^ μm^2^ in C57BL/6 (Fig. 4A–a), 18.1 ± 2.5 cells/10^4^ μm^2^ in N-Shc+/+ mice (Fig. 4A–b), and 43.5 ± 4.2 cells/10^4^ μm^2^ in N-Shc−/− mice (Fig. 4A–c). There was a significantly larger number of pyramidal cells in the CA3 area in N-Shc−/− mice as compared to C57BL/6 and N-Shc+/+ mice on day 1 after KA administration (p < 0.001, Fig. 4A–d).

On day 7, the number of pyramidal cells in the CA3 region was15.5 ± 5.9 cells/10^4^ μm^2^ in C57BL/6 mice (Fig. 4A–e), 20.2 ± 4.4 cells/10^4^ μm^2^ in N-Shc+/+ mice (Fig. 4A–f), and 45.0 ± 2.6 cells/10^4^ μm^2^ (Fig. 4A–g). There was a significantly larger number of pyramidal cells in the CA3 area in N-Shc−/− mice as compared to C57BL/6 and N-Shc+/+ mice on day 7 after KA administration (p < 0.001, Fig. 4A–h).

Thus, neuronal cell loss was observed in the pyramidal cell layer of the CA3 area in C57BL/6 and N-Shc+/+ mice on both days 1 and 7 after KA-administration. In contrast, less cell loss was seen in the CA3 area in N-Shc−/− mice on both days 1 and 7 after KA administration.

In contrast to CA3, the pyramidal cells in the CA1 area were unaffected in all of the three groups on days 1 and 7 after KA administration (Fig. 4B).

## Discussion

In the present study, we used a mouse model of KA-induced seizures to examine whether N-Shc, downstream signal adaptor of TrkB, play a role in epileptiform activity. We observed the following results: (1) in the N-Shc−/− mice, KA-induced behavioral seizures were less severe and the frequency of the epileptiform discharges was lower as compared with controls; and (2) KA-induced neuronal cell loss in the pyramidal cell layer of CA3 was greatly reduced in N-Shc−/− mice. Together, these results suggest that the activation of the N-Shc-mediated signaling pathway contribute to the acute epileptiform activity induced by KA.

Previous studies have shown that the activation of BDNF signaling is closely associated with epileptogenesis^20^. The data from the present study are in agreement with past studies of BDNF expression. These have shown that the occurrence of seizures leads to an increase of the expression level of BDNF mRNA and protein in the hippocampus^4^,^5^,^21^, and that the increased expression of BDNF results in both hyper-excitability and the reduction of inhibitory synaptic transmission in hippocampal neurons^9^,^22^,^23^,^24^. The data from the present study similarly suggest that the BDNF system plays a role in neuronal hyper-excitation.

Treatments with either TrkB-Fc or K252a, an inhibitor of the TrkB receptor, have reduced KA-induced epileptiform activity and the development of kindling in rats^6^,^25^. Interestingly, the conditional deletion of the TrkB receptor prevents epileptogenesis in the mouse model of kindling^26^. Reductions in electrophysiological measurements and the absence of behavioral evidence of epileptogenesis were observed in TrkB−/− mice. Inhibition of TrkB kinase activity exerted antiseizure effect in the kindling mice model or following KA induced status epilepticus^8^,^27^. Intriguingly, these authors have also shown that no detectable regulatory effects on kindling were observed in mice carrying a point mutation in the Shc binding site (Y515F) of TrkB^17^. In contrast, kindling was partially inhibited in mice with a point mutation in the PLCγ binding site of TrkB^18^. Treatment with a peptide pY816, uncoupling TrkB from PLCγ, following KA induced status epilepticus inhibited spontaneous seizures^19^. These studies support the notion that BDNF-TrkB signaling via PLCγ, rather than N-Shc, controls limbic epileptogenesis through the positive regulation of excitatory synaptic transmission.

Expression of KCC2 is also decreased in the mouse hippocampus after kindling, and the reduction of KCC2 expression can be prevented by inhibiting TrkB signaling, which recruits both N-Shc and PLCγ-coupled signaling pathways^28^. These data suggest that TrkB-mediated signaling is essential for kindling-induced limbic epileptogenesis, potentially through the regulation of synaptic neurotransmission.

Given the inverse relationship between TrkB-mediated signaling and the expression of KCC2, a positive regulator for GABAergic neurotransmission^10^,^28^, it would be beneficial to gain a better understanding of TrkB-mediated signaling for the development of therapeutic approaches to control epilepsy.

In the present study, we demonstrated that the deficiency in N-Shc led to a reduction in both the severity of behavioral seizures and epileptiform discharges in the hippocampus of the KA-induced acute seizure model. These results, therefore, suggest that the N-Shc-mediated signaling pathway is contributes to the acute epileptiform activity induced by KA. However, we have not evaluated the roles of TrkB and N-Shc-mediated signaling in the development of kindling or of spontaneous seizures following KA-induced status epilepticus, i.e., epileptogenesis. This might be the subject of future experiments.

Elimination of TrkB or the acute inhibition of TrkB function impairs the induction of long-term potentiation (LTP) at the synapses of CA3 mossy fibers^29^ and Schaffer collateral-CA1 circuitries^30^. It has also been suggested that the TrkB-PLCγ signaling pathway is necessary for the maintenance of LTP^31^,^32^,^33^. For therapeutic purposes, the inhibition of TrkB might prevent seizures. However, the inhibition of TrkB would also affect the PLCγ-mediated signaling pathway, and consequently, long-term memory and cognition as a result of impairing LTP. Intriguingly, our previous study has demonstrated that N-Shc−/− mice have superior cognitive function associated with enhanced LTP in the hippocampus^16^. Together with the present study, our data imply that suppression of N-Shc-mediated signaling, not via PLCγ, at the downstream of TrkB could reduce epileptiform activity without affecting long-term memory storage and cognition. Therefore, N-Shc itself and/or its downstream components could be potential therapeutic targets for the treatment of epilepsy.

In our study, stereological analysis of neuronal cell loss in area CA1 and CA3 of hippocampus was not performed. However, the patterns of seizure-induced cell loss in the control C57BL/6 and N-Shc+/+ mice were similar to those observed in other studies using C57BL/6 mice model of TLE with/without stereological analysis^34^,^35^,^36^. In the future, the stereological analysis for our KA-induced seizures model in the N-Shc−/− mice would be applied for the accurate evaluation.

## Conclusions

This study demonstrates that the activation of the N-Shc-mediated signaling pathway contributes to an acute KA-induced epileptiform activity and neuronal cell loss in the hippocampus. Future studies will be necessary to clarify whether N-Shc acts solely downstream of BDNF-TrkB and/or other signaling components. Further investigations are also needed to understand the molecular mechanism by which N-Shc-mediated signaling modulates synaptic transmission and maintains neuronal hyperexcitability during behavioral seizures and epileptogenesis. Nonetheless, our study has provided the first evidence that N-Shc and its downstream signaling components could be novel therapeutic targets for the management of epilepsy, preventing epileptogenesis and/or limiting its progression.

## Methods

### Experimental animals

For this experiment, we used 4–8 week-old male N-Shc homozygous mutant mice(N-Shc−/−). N-Shc−/− mice were originally generated by Sakai *et al*^37^. and subsequently backcrossed with C57BL/6J over 13–15 generations as described previously^16^. We have previously reported that in N-Shc−/− mice the gross anatomy of the hippocampus -as well as other brain regions such as the cerebral cortex and thalamus where N-Shc is highly expressed- are normal, and that their development is the same as in wild-type mice^14^,^16^. The input-output relationship of synaptic transmission in the hippocampus has also been examined by measuring the amplitude of the presynaptic fiber volleys(PSFVs), excitatory post synaptic potentials(EPSPs) and paired-pulse facilitation(PPF) in N-Shc−/− and wild-type mice. These studies have shown that there is no deterioration of synaptic transmission in N-Shc−/− mice, as compared with wild-type mice^16^. The genotypes of the mice were determined by PCR analyses of their tail DNAs. In addition, 4–8 week-old male wild-type littermates of N-Shc homozygous mutant mice(N-Shc+/+) and C57BL/6 mice were used as controls. The mice were housed in plastic cages in a regulated environment (24 ± 1 °C; 50 ± 5% humidity) with a 12 hour light/dark cycle (lights on at 7:00 am). Food and tap water were available ad libitum. All experiments were carried out according to the guidelines and approved by the Animal Welfare Committee of Nagasaki University. Efforts were made to minimize animal suffering and to reduce the number of animals used.

### Western blot analysis

Mice were sacrificed by decapitation under deep anesthesia with diethylether. Following decapitation, the brains were harvested and dissected microsurgically into three sections:1)the whole hippocampus, 2)a part of the cerebral cortex(dorsal part), and 3)a part of the thalamus(dorso-medial part). Each dissected region was homogenized in lysis buffer(50 mM Tris-HCl, pH 8.0/150 mM NaCl/1.0% NP-40/0.5% sodium deoxycholate/0.1% SDS/0.5 mM PMSF/10 mM NaF/2 mM Na3VO4/1X complete protease inhibitor cocktail; Roche Diagnostics GmbH). Samples containing equal amounts of protein were mixed with 2X sample buffer and heated for 1 min at 95 °C. The protein samples were analyzed 10% SDS–PAGE for 1.5 hours at 125 V and then transferred onto polyvinylidene difluoride membranes(GE Healthcare) for 1 hour at 25 V. The membranes were blocked with 5% blocking agent in 1X Tris-buffered saline/Tween-20 buffer for 1 hour at room temperature and subsequently incubated with primary antibodies at 4 °C overnight. The membranes were rinsed with Tris-buffered saline/Tween-20 and incubated with secondary antibodies for 1 hour at room temperature. The signals were detected with ECL Plus reagent(GE Healthcare) on X-ray films. The primary antibodies used were mouse anti-ShcC antibody(1:5000; BD Transduction Laboratories), anti-mouse TrkB antibody(1:1000; R&D Systems, Inc.), anti-GluR6(1:200; Santa Cruz Biotechnology, Inc.), anti-KA1(1:200; Santa Cruz Biotechnology, Inc.), anti-KA2(1:200; Santa Cruz Biotechnology, Inc.) and anti-β-Actin(1:5000; Sigma Aldrich, Inc.). To quantify protein expression, the film was scanned and the immunoreactivity of individual band intensity on Western blots was measured by ImageJ 1.46r software(Wayne Rasband, National Institutes of Health) and normalized to β-actin content. The value for each protein band of sample was calculated as mean.

### KA treatment and assessment of behavioral seizures

For the induction of behavioral seizures, the mice (C57BL/6, N-Shc+/+, and N-Shc−/−) received a single intraperitoneal(i.p.) administration of KA(Kainic Acid Monohydrate, Wako Pure Chemical Industries, Ltd.) dissolved in physiological saline(6 mg/ml) at a dose of 30 mg/kg bodyweight. A 30 mg/kg dose of KA administration(i.p.) was used in all of the following experiments, as it caused neuronal cell loss in the hippocampal CA3 area, which is a feature reminiscent of human TLE. Lower KA doses did not consistently produce cell loss.(see Supplementary Table S1).

The mice were continuously monitored for 320 min to register the onset and extent of the seizure activity. The behavioral seizures were evaluated every 5 min, and the maximum seizure score at each period of 5 min was recorded. The behavioral manifestations of the seizures were classified according to Racine^38^: (1)mouth and facial clonus; (2)head nodding; (3)forelimb clonus; (4)rearing; (5)rearing, falling (full motor seizure with loss of postural control) and status epilepticus and (6)death were also recorded. The duration of the behavioral seizures after KA administration was measured from the time of starting Racine scale1 to the time of end of seizures or death.

### Hippocampal EEG recording

The mice (C57BL/6 and N-Shc−/−) were anaesthetized with sodium pentobarbital(45 mg/kg) and placed in a stereotaxic frame in the flat skull position. An incision was made at the midline of the scalp to expose the skull, burr holes were drilled above the right and left hippocampal areas, and the dura was pierced and removed. Teflon-coated, stainless steel monopolar electrodes 0.2 mm in diameter, insulated except for the tip, were implanted in both hippocampi close to the CA3 region using the following coordinates with bregma as the reference: 2.0 mm posterior, 2.25 mm lateral, and 2.25 mm below dura^39^. As a reference electrode, an epidural screw was implanted into the skull over the frontal cortex 2.0 mm anterior to the bregma, and a ground electrode was implanted over the cerebellum. All electrodes were connected to a socket and fixed to the skull with dental acrylic resin. The mice were allowed to recover for 7 days after surgery prior to starting the electroencephalogram(EEG) recordings. After recovery, behavior and EEG of the mice was observed in clean cage. The EEG was amplified and filtered below 0.3 and above 30 Hz on Bioelectric Amp (UA-102, Unique Medical Co, Ltd.). The baseline hippocampal EEG was recorded for at least 30 min. Thereafter, behavioral seizures were induced by KA administration (30 mg/kg,i.p.) in each mouse. EEGs were recorded continuously for 2 hours after the KA treatment. Digitally recorded EEG files were analyzed by browsing the EEG manually on a computer screen. Epileptiform discharges, spikes and sharp waves, were defined as high-amplitude (>2 × baseline) and frequency (>5 Hz) discharges in the hippocampus that lasted for a minimum of 5 seconds. The number of epileptiform discharges was counted within 1 hour and 1 to 2 hours after KA treatment in each animal. The mean frequency (number of epileptiform discharges/hour) was calculated in each group.

### Histology and hippocampal cell morphology

On day1(24 hours) and day7 after seizure induction by KA (30 mg/kg,i.p.), the mice (C57BL/6, N-Shc+/+, and N-Shc−/−) were deeply anesthetized with diethylether and transcardially perfused, first with phosphate-buffered saline (PBS) and then with 4% paraformaldehyde in a 0.1 M phosphate buffer (pH7.4). The brains were harvested, post-fixed with 4% paraformaldehyde for 1 hour and cryoprotected in 30% sucrose for at least 12 hours. The brain tissues were embedded into optimal cutting temperature(OCT) compound and stored at −80 °C until sectioning. 10-μm coronal sections were then cut with a freezing microtome. The consecutive sections were cut throughout the entire hippocampus from the rostral to the caudal direction using the following coordinates with bregma as the reference: from 1.0 mm posterior to 3.0 mm posterior. The sections were mounted onto microscope slides and air-dried. The mounted sections were rinsed with distilled water to remove the OCT compound and immersed in a 0.1% cresyl violet (Nissl) solution for 5 min. Subsequently, the slides were dehydrated through an ascending series of ethanol solutions, cleared in xylene, and coverslipped using Permount.

Only the dorsal hippocampal region showed obvious morphological changes throughout the entire hippocampus after KA administration(30 mg/kg,i.p.). Therefore we quantified neuronal cell loss in the CA1 and CA3 regions of the dorsal hippocampus. We studied coronal sections at 10-μm thickness for the dorsal hippocampus from 1.5 mm to 2.5 mm posterior with bregma as the reference coordinates. We selected 3 different slice sections of the dorsal hippocampus separated by 100 μm in each mouse. Images (TIFF files) of the Nissl stained slice sections were captured using an AxioCam HRc camera with an Axioskop 2 plus microscope(Carl ZEISS) at 100x magnification. The captured images were loaded into Image-J software to analyze hippocampal cell morphology after seizure induced by KA. Neuronal cell loss was quantified in the pyramidal cell layer of CA1 and CA3 regions of the dorsal hippocampus. The number of pyramidal cell nuclei was visually counted within the 3 distinct segments of 100 × 100 μm square fields for each CA1 and CA3 region.(see Supplementary Fig. S1 online) The mean number of pyramidal cell nuclei (number of cells/10^4^ μm^2^) within the 3 segments was calculated in the 3 slice sections for each mouse. We compared the mean number of pyramidal cell nuclei of CA1 and CA3 region for 3 groups (C57BL6, N-Shc+/+ and N-Shc−/−) on day1 and day7.

### Statistical analysis

Statistical analyses were performed using SPSS ver.15.0(SPSS, Inc.). Comparisons were made using the Student’s two-tailed unpaired t-test, when appropriate. The seizure scores were compared using the Mann-Whitney test. The data were presented as the mean ± standard deviation(SD). P values of less than 0.05 were considered statistically significant.

## Additional Information

**How to cite this article**: Baba, S. *et al*. Involvement of the neuronal phosphotyrosine signal adaptor N-Shc in kainic acid-induced epileptiform activity. *Sci. Rep*. **6**, 27511; doi: 10.1038/srep27511 (2016).

## Supplementary Material

Supplementary Information

## Figures and Tables

**Table 1:** Maximum seizure scale and duration of seizure among 3 groups.

Genotype	KA dose (mg/kg)	Maximum seizure scale	Duration of seizure (min)
C57BL6	30	5.21 ± 0.98	167.2 ± 63.8
N-Shc+/+	30	5.50 ± 0.71	172.1 ± 24.6
N-Shc−/−	30	3.27 ± 1.78*	95.3 ± 32.6*

Mean maximum seizure scale and mean duration of seizure were significantly decreased in N-Shc–/– mice compare to N-Shc+/+ and C57BL/6 mice (*p < 0.001).
