# Supplementary material for: Involvement of the neuronal phosphotyrosine signal adaptor N-Shc in kainic acid-induced epileptiform activity
Source: Sci Rep. 2016 Jun 8;6:27511. doi: 10.1038/srep27511 (PMC4897738; doi:10.1038/srep27511)
Supplement: Supplementary Information [file srep27511-s1.pdf]

Involvement of the neuronal phosphotyrosine signal adaptor N-Shc in kainic acid-induced epileptiform activity

Shiro Baba[1,2,3,*], Kazuko Onga[1], Sho Kakizawa[1], Kyoji Ohyama[1], Kunihiko Yasuda[1], Hiroshi Otsubo[3], Brian W. Scott[4], W. McIntyre Burnham[4], Takayuki Matsuo[2], Izumi Nagata[2] and Nozomu Mori[1,*]

Supplementary Figure S1

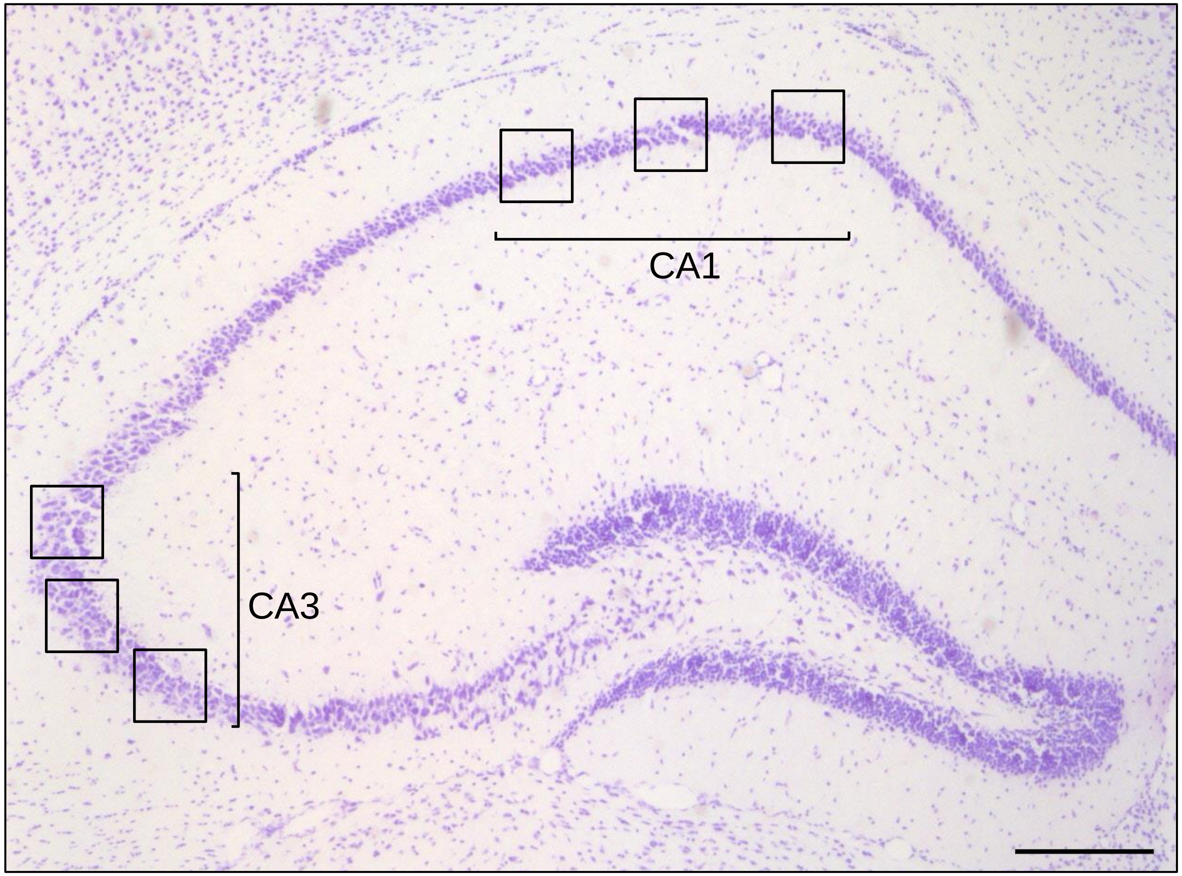

Evaluation of cell loss in the pyramidal cell layer of the dorsal hippocampus
The number of pyramidal cell was counted within the 3 distinct segments of 100x100 µm square fields for each CA1 and CA3 region. Scale bars=200µm.

Supplementary TableS1

Dose of kainic acid (KA) and hippocampal neuronal cell loss in C57BL/6 mice

KA dose (mg/kg i.p.)	Maximum seizure scale	Neuronal cell loss
15	4.0±0	-
20	4.7±0.76	-
30	5.2±0.79	+
